# Exploring polar headgroup interactions between sphingomyelin and ceramide with infrared spectroscopy

**DOI:** 10.1038/s41598-020-74781-8

**Published:** 2020-10-19

**Authors:** Igor de la Arada, Emilio J. González-Ramírez, Alicia Alonso, Félix M. Goñi, José-Luis R. Arrondo

**Affiliations:** Instituto Biofisika (CSIC, UPV/EHU), and Departamento de Bioquímica, Universidad del País Vasco, 48940 Leioa, Spain

**Keywords:** Biochemistry, Lipids, Sphingolipids, Biophysics, Membrane structure and assembly

## Abstract

Ceramide is a major actor in the sphingolipid signaling pathway elicited by various kinds of cell stress. Under those conditions ceramide (Cer) is produced in the plasma membrane as a product of sphingomyelin (SM) hydrolysis, and this may lead to apoptosis. Thus, SM and Cer coexist in the membrane for some time, and they are known to separate laterally from the (more abundant) glycerolipids, giving rise to highly rigid domains or platforms. The properties of these domains/platforms are rather well understood, but the underlying SM:Cer molecular interactions have not been explored in detail. Infrared (IR) spectroscopy is a powerful analytical technique that provides information on all the chemical groupings in a molecule, and that can be applied to membranes and lipid bilayers in aqueous media. IR spectra can be conveniently retrieved as a function of temperature, thus revealing the thermotropic transitions of SM and its mixtures with Cer. Four regions of the IR spectrum of these sphingolipids have been examined, two of them dominated by the hydrophobic regions in the molecules, namely the C–H stretching vibrations (2800–3000 cm^−1^), and the CH_2_ scissoring vibrations (1455–1485 cm^−1^), and two others arising from chemical groups at the lipid-water interface, the sphingolipid amide I band (1600–1680 cm^−1^), and the phosphate vibrations in the 1000–1110 cm^−1^ region. The latter two regions have been rarely studied in the past. The IR data from the hydrophobic components show a gel (or ripple)-fluid transition of SM at 40 °C, that is shifted up to about 70 °C when Cer is added to the bilayers, in agreement with previous studies using a variety of techniques. IR information concerning the polar parts is more interesting. The amide I (carbonyl) band of pure SM exhibits a maximum at 1638 cm^−1^ at room temperature, and its position is shifted by about 10 cm^−1^ in the presence of Cer. Cer causes also a change in the overall band shape, but no signs of band splitting are seen, suggesting that SM and Cer carbonyl groups are interacting tightly, presumably through H-bonds. The 1086 cm^−1^ band, corresponding to PO_2_^−^ vibrations, appears more stable in SM than in DPPC, and it is further stabilized by Cer, again suggesting an important role of H-bonds in the formation of SM:Cer clusters. Thus, SM and Cer can interact through their polar headgroups, in a way that is not accessible to other lipid classes.

## Introduction

Sphingolipids are important components of cell membranes^1^. Among them, sphingomyelin (SM) plays a structural role, stabilizing the lamellar structure, and is also a major source of ceramide (Cer) in the plasma membrane^2^. Under cellular stress conditions SM is hydrolyzed by shingomyelinases to Cer and the water-soluble phosphorylcholine. Then Cer elicits the sphingolipid signaling pathway, leading to the programmed cell death or apoptosis^2,3^. In addition to its role as a metabolic signal, Cer perturbs in various ways the membrane bilayer architecture and properties^4^. SM and Cer have been proposed to interact strongly through hydrogen bonding^5–8^, thus it can be reasonably assumed that, under stress conditions, SM:Cer clusters or complexes are formed in the cell plasma membrane^2^.

SM:Cer mixtures have been studied using a variety of techniques, such as fluorescence microscopy^7,9–11^, differential scanning calorimetry^7,12^, lipid monolayers^13,14^, atomic force microscopy^15–17^, D-NMR^18^, or X-ray scattering^19^. These studies have revealed a host of interesting properties of the above mixtures which, among other things, may constitute the only (micro)domains existing in the gel phase in cell membranes^20^.

In the present study we have further explored the SM:Cer system using IR spectroscopy. This technique provides very detailed, separate information from each of the chemical groupings making up the lipid molecules, in an aqueous environment^21^. Bilayers consisting of SM in mixtures with other phospholipids and cholesterol have been studied by IR spectroscopy^22,23^. The technique has also been applied to ceramides in mixtures mimicking the skin stratum corneum^24,25^. Boulgaropoulos et al.^19^ combined IR, calorimetric and X-ray diffraction techniques to describe ternary mixtures of SM, Cer and phosphatidylcholine. Both egg SM (eSM) and palmitoyl SM (pSM) have been used in our study. eSM contains about 80% palmitoyl SM. Recent X-ray studies on oriented thick bilayer stacks^26^ have revealed that eSM exists in a ripple phase between 3 and 38 ºC, and it undergoes a transition to the fluid phase at the latter temperature. eSM, in turn, was in a gel phase at 3 °C, with a gel-to-ripple transition at ∼ 24 °C and a ripple-to-fluid transition at ∼ 41 °C.

Our IR study of SM:Cer mixtures provides information on this deceptively simple, but in practice complex lipid system, and our results can shed light on the unique properties of the SM and Cer sphingosine-based headgroups that are not found in glycerolipids.

## Materials and methods

### Materials

Hen egg sphingomyelin (eSM, 860061), N-palmitoyl-d-erythro-sphingosylphosphorylcholine (palmitoyl sphingomyelin, pSM, 860584), and egg ceramide (Cer, 860051) were purchased from Avanti Polar Lipids (Alabaster, AL, USA). D_2_O was purchased from Merck (Darmstadt, Germany). All other reagents (salts and organic solvents) were of analytical grade. Buffer solution for liposome preparation was 20 mM PIPES, 1 mM EDTA, 150 mM NaCl, pH 7.4.

### Liposome preparation

All lipid mixtures are given as mole ratios. Lipid vesicles were prepared essentially as described in^7^. The desired lipids dissolved in chloroform/methanol (2:1, v/v) were mixed and the solvent was evaporated under a stream of nitrogen. The lipid film was kept under high vacuum for 90 min to ensure the removal of undesired organic solvent. Multilamellar vesicles (MLV) were formed by hydrating the lipid film with the buffer solution at 90 °C, helping the dispersion with a glass rod. The samples were incubated for 10 min in a bath sonicator at the same temperature, to facilitate homogenization. For IR analysis the vesicle suspension was freeze-dried and the resulting dehydrated sample was resuspended in D_2_O, with vortexing and forcing the suspension through a narrow pipette.

### Infrared spectroscopy

Infrared spectra were recorded in a Thermo Nicolet Nexus 5700 (Thermo Fisher Scientific, Waltham, MA) spectrometer equipped with a liquid nitrogen-refrigerated mercury-cadmium-telluride detector using a Peltier-based temperature controller (TempComp, BioTools Inc., Wauconda, IL). A 25-μl sample aliquot was deposited on a 25 μm optical path calcium fluoride cell (BioCell, BioTools Inc., Wauconda, IL) that was sealed with a second cell. Typically, 370 scans for each, background and sample, were collected at 2 cm^−1^ resolution and averaged after each minute. Temperature was increased at a rate of 1 °C/min. Data treatment and band decomposition of the original amide I have been described elsewhere^27^.

## Results

Fully hydrated samples of pure SM (eSM or pSM), or of eSM:Cer mixtures at 85:15 or 70:30 mol ratios, were studied by IR spectroscopy as a function of temperature. The thermotropic properties of these samples, as derived from differential scanning calorimetry (DSC) studies, are shown in the Supplementary Fig. S1 and in Table 1. The calorimetric data were used as a guide for IR studies.

**Table 1:** Onset (T_s_) and completion (T_f_) temperatures (in ºC) for the gel-fluid transitions of SM and SM:Cer mixtures.

Sample	Technique	T_s_	T_f_	Refs.
eSM	DSC	36	40	^7^
eSM:Cer (90:10)	DSC	34	55	^7^
eSM:Cer (70:30) DSC	33	63	7	
pSM	DSC	39	41	^12^
pSM:Cer (90:10)	DSC	40	64	^12^
pSM:Cer (70:30)	DSC	48	71	^12^
eSM	IR 2918	38	42	This paper
eSM:Cer (85:15)	IR 2918	56	65	This paper
eSM:Cer (70:30)	IR 2918	63	72	This paper
pSM	IR 1467	41	53	This paper
eSM:Cer (85:15)	IR 1467	48	76	This paper
eSM:Cer (70:30)	IR 1467	60	72	This paper
eSM	IR 1632	31	45	This paper
eSM:Cer (85:15)	IR 1632	46	63	This paper
eSM:Cer (70:30)	IR 1632	59	74	This paper
pSM	IR 1632	39	45	This paper
pSM	D-NMR	39	41	^18^
pSM:Cer (90:10)	D-NMR	39	70	^18^
pSM:Cer (70:30)	D-NMR	38	75	^18^

Compositions are given as mol ratios. Temperatures are rounded off to the nearest integer. Averages of 2 closely similar experiments.

**Figure 1:**
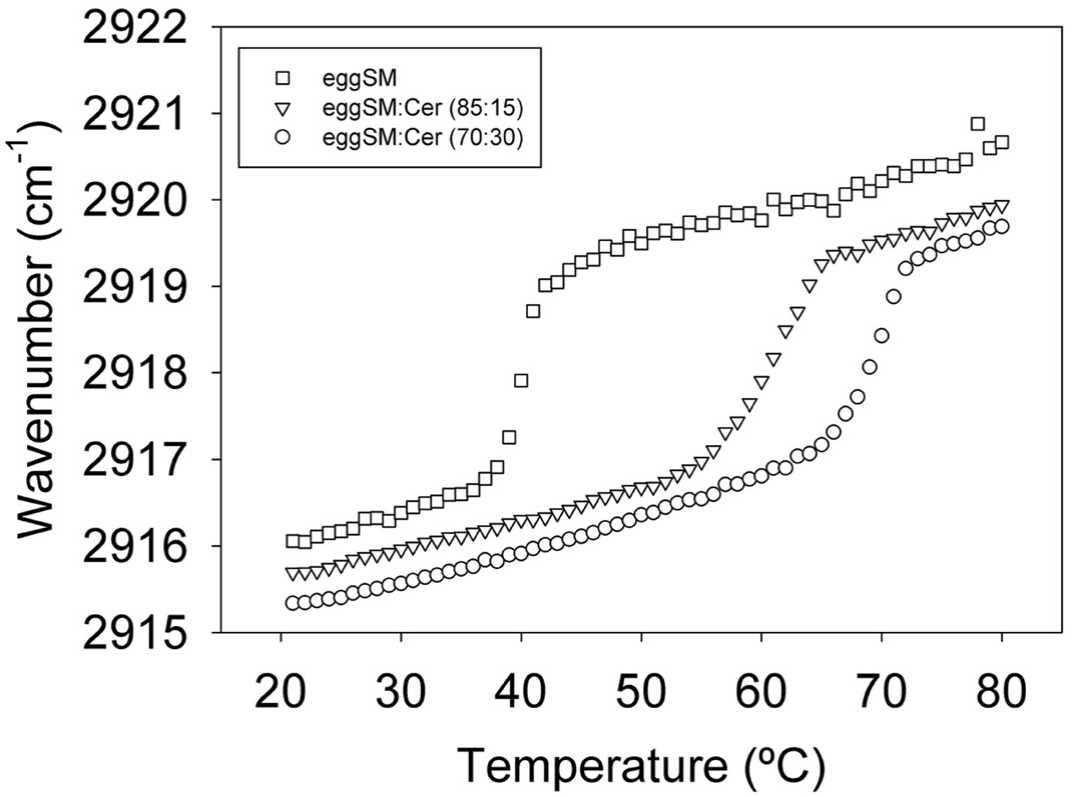
Asymmetric C-H stretching vibrations in the IR spectra of aqueous dispersions of pure eSM and eSM:Cer mixtures. Band maxima as a function of T. Squares, eSM. Triangles, eSM:Cer (90:10). Circles, eSM:Cer (70:30).

The 2870–2950 cm^−1^ region of the phospholipid IR spectrum corresponds to the asymmetric C-H stretching vibrations, with maxima at 2915–2921 cm^−1^^28,29^ (Fig. S2A,E). Figure 1 shows plots of the asymmetric C–H stretching band maxima as a function of temperature, for the three eSM-based samples. A steep increase in band position revealed a ripple-to-fluid phase transition of the lipid mixture. The data for pure eSM corresponded well with previously published results^22,23^, and were in good correlation with the calorimetric studies (further inter-technique comparisons are dealt with in the Discussion). The presence of Cer widened the transition temperature range and shifted the transition to higher T (Table 1). The behavior of pure pSM and eSM was very similar, as shown in the Supplementary Fig. S3A,F, in spite of the differences in the ordered phases observed by Arsov et al.^26^. The band assigned to the symmetric C-stretching vibrations (maxima at 2846–2851 cm^−1^) behaved similarly to the asymmetric one in what refers to the transition onset temperature T_s_, but the shift to lower wavenumbers was less steep, and suggested a somewhat wider transition temperature range (Fig. S3A,B,F,G).

**Figure 2:**
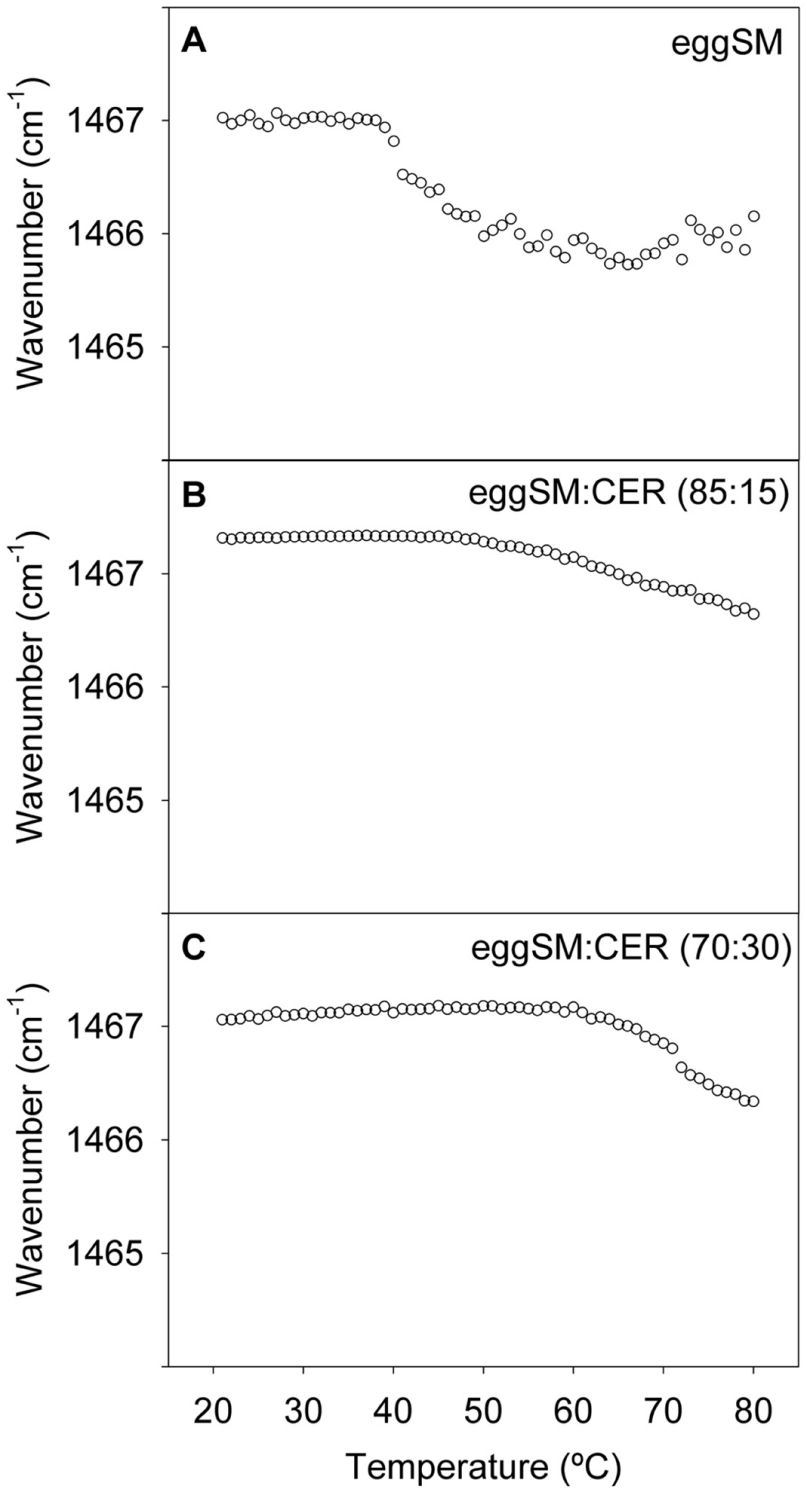
CH_2_ scissoring vibrations in the IR spectra of aqueous dispersions of pure eSM and eSM:Cer mixtures. Band maxima as a function of T. (**A**) eSM. (**B**) eSM:Cer (90:10). (**C**) eSM:Cer (70:30).

The 1455–1485 cm^−1^ IR spectral region is usually assigned to methylene scissoring vibrations. A split band at low T in this region has been interpreted as indicating an orthorhombic chain packing in the membrane^24,25^, but in our case single bands were observed under all conditions (Supplementary Fig. S2B,F). Band maxima were plotted as a function of T in Fig. 2. For pure eSM an abrupt change in position was seen at the ripple-fluid transition. In the presence of Cer though the shift was less abrupt and it occurred at higher T, in agreement with the calorimetric data (Table 1). pSM exhibited a behavior very similar to that of eSM (Supplementary Fig. S3C,H).

**Figure 3:**
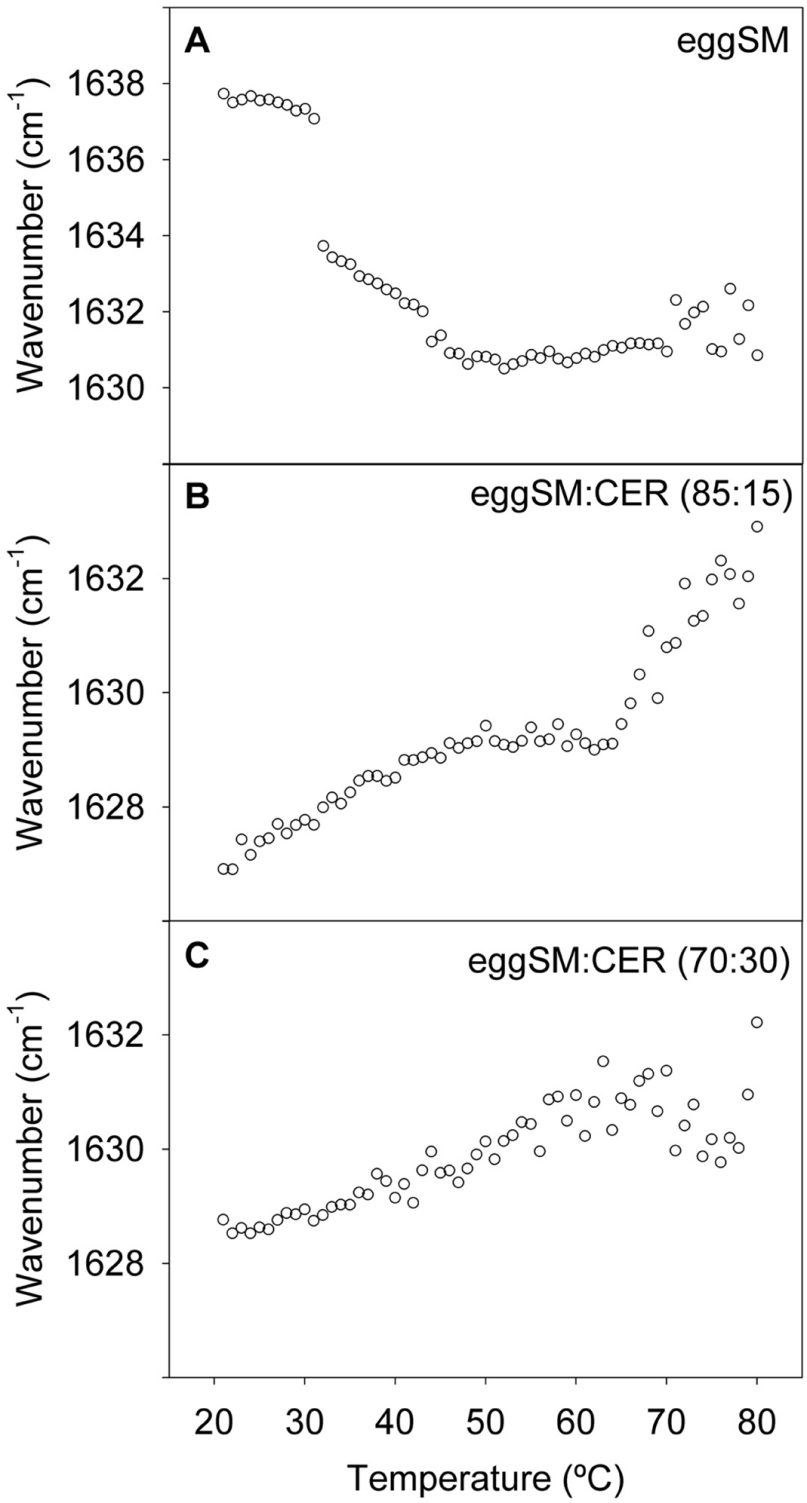
Amide I band in the IR spectra of aqueous dispersions of pure eSM and eSM:Cer mixtures. Band maxima as a function of T. (**A**) eSM. (**B**) eSM:Cer (90:10). (**C**) eSM:Cer (70:30).

SM is unique among the common phospholipids in that it contains an amide group, which originates from a fatty acid linked to the amino group in C2 of sphingosine^6^. The vibrational signal maximum from SM amide C=O (amide I band) occurs around 1630–1640 cm^−1^^19^. The corresponding plot as a function of T is shown in Fig. 3A. With pure SM (both pSM and eSM, see Fig. S3D,I) increasing temperatures led to a shift towards lower wavenumbers. For eSM, a sharp shift at ≈ 30 °C was followed by a more gradual displacement in the 30–45 °C interval, down to ≈ 1631 cm^−1^. In this case pSM behaved differently, with a gradual shift in the 25–40 °C range and a sharp shift between 40 and 50ºC, followed by a slight upward shift (Fig. S3D,I). Note that lipid phase transitions, as recorded with the usual techniques (DSC, wide-angle X-ray scattering, and so on), reflect mainly changes in the phospholipid acyl chains, while the 1630–1640 cm^−1^ signal arises from a structure in the lipid polar headgroup, and this may account for the observed differences in thermal behavior between the C–H and CH_2_ vibrations (Figs. 1, 2) and the C=O signal in Fig. 3. In general, changes in the phospholipid headgroups accompanying the gel/ripple-fluid transitions had not been studied in detail in the past.

Cer contains as well an amide group, of the same chemical origin as SM. Cer is too hydrophobic to allow retrieval of IR spectra of pure Cer in aqueous dispersion. However the presence of Cer in the SM bilayers modified considerably the thermal properties of the SM amide band (Fig. 3B,C), giving rise to a mixed SM + Cer band. At low T, the band maximum appeared at ≈ 1628 cm^−1^ (about 10 cm^−1^ lower than the pure eSM) and it was shifted towards *higher* wavenumbers with increasing T. With 15 mol% Cer the shift reached a plateau at ≈ 48 °C, and it rose again steeply above 63 °C. With 30 mol% Cer the T-dependent shift occurred rather steadily, at least up to 70 °C. At 70–80 °C, when they were in the fluid state, all three samples had their maxima at ≈ 1631 cm^−1^.

**Figure 4:**
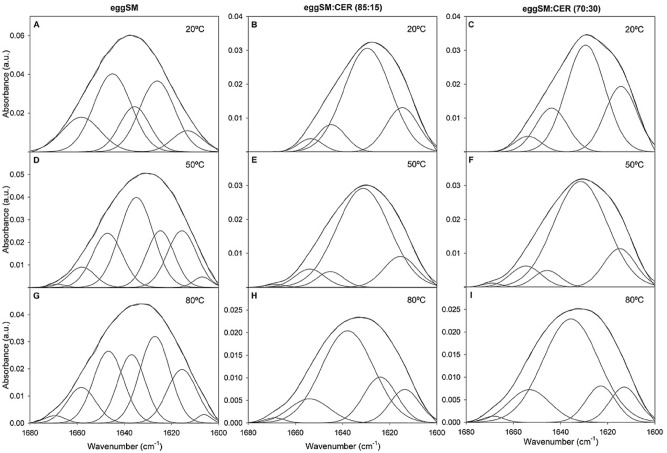
The amide I band region (1600–1680 cm^−1^) of the IR spectrum of aqueous dispersions of pure eSM and eSM:Cer mixtures. Compositions and temperatures are indicated for each panel.

The SM (or SM + Cer) amide I band (≈ 1600–1680 cm^−1^) did not only change in position with temperature and with the presence of Cer, it also showed changes in shape, becoming less symmetric in the presence of Cer. This would point to the existence of populations differing in the interaction of the amide group with the environment. Figure 4 exemplifies the shape and position of that band, at 20, 50 and 80 °C, i.e. below, during, and above the gel-fluid phase transition, for pure SM, and for SM:Cer 85:15 and 70:30 mixtures (mol ratios). The overall shift towards lower wavenumbers (frequencies) in the presence of Cer is generally interpreted as stronger H-bonded species^30^. Additional information can be obtained from fitting the amide band with its spectral components, following a procedure developed for amide I bands of proteins^31^. Figure 4 includes, for each amide band, the minimum number of band components that fitted closely the band envelope. The corresponding spectral regions for pure SM are shown in Fig. 4A,D,G, at 20, 50, and 80 °C respectively. Since sufficiently noise-free data, allowing e.g. to continuously follow the shifts in each band component position as a function of temperature, were not available, obtaining additional information on the amide I band from fitting would require an improvement in data retrieval. However, even with those limitations, a series of reliable observations could be made from amide I band fitting. A minimum number of 5 components was required for a good fitting of the pure SM amide I band at 20 °C. These components could not be properly assigned with our present level of knowledge. They might correspond to SM molecules with different orientations and/or degrees of H-bonding, meaning that SM would not be in the crystalline state, rather the data would be suggesting the presence of a hexatic phase, in which dislocations occur^32^. When T was increased to 50 and 80 °C, the number of components increased to 6 and 7 respectively (Fig. 4D,G). This would be consistent with a transition from a hexatic to a liquid-crystalline phase, in which heterogeneity in H-bonding would be favored not only by dislocations but also by disclinations^33^. In the presence of Cer (Fig. 4B,C,E,F,H,I) a prominent band component was seen with a maximum at ≈ 1630 cm^−1^, this component being the chief cause for the SM + Cer amide band shift to lower wavenumbers in the presence of Cer. Increasing temperatures did not have a large effect on the SM + Cer amide band, except for a novel component appearing at ≈ 1612 cm^−1^ at 80 °C, i.e. when the system was in the fluid phase. Even if a reliable assignment is not feasible, it should be mentioned that Boulgaropoulos et al.^19^ found, in the amide I band of a POPC:SM:Cer mixture, a component at ≈ 1616 cm^−1^ that they assigned to “pure, H-bonded Cer”.

**Figure 5:**
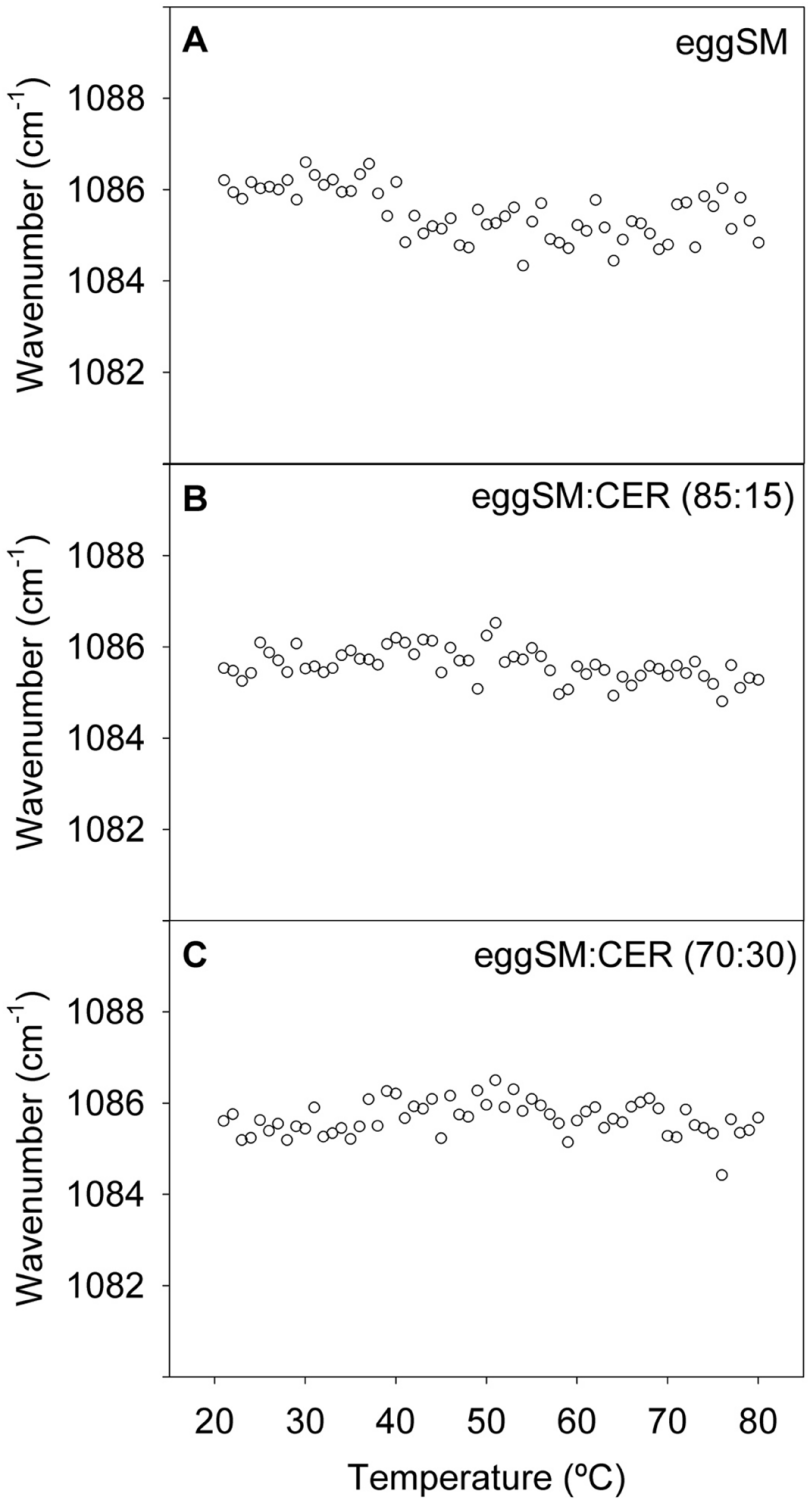
Symmetric PO_2_ stretching vibrations in the IR spectra of aqueous dispersions of pure eSM and eSM:Cer mixtures. Band maxima as a function of T. (**A**) eSM. (**B**) eSM:Cer (90:10). (**C**) eSM:Cer (70:30).

The 1000–1300 cm^−1^ spectral region, corresponding to phosphate group vibrations, remains virtually unexplored. In an early paper, Arrondo et al.^34^ assigned three bands in that region to various vibrational modes of the DPPC phosphate group, with maximum wavenumbers at 1060, 1086 and 1222 cm^−1^. The latter two were assigned, respectively, to symmetric and asymmetric PO_2_^−^ stretching, while a shoulder at 1060 cm^−1^ was attributed to an R–O–P–O–R’ stretching mode, where both phosphate ester substituents are different. For SM (Fig. S2) the best resolved maximum is the one at 1086 cm^−1^, which we have chosen for our investigation. (The spectral region above 1120 cm^−1^ has not been considered in the present study because it is perturbed by the D_2_O used as solvent). The effect of temperature on the 1086 cm^−1^ band position is shown in Fig. 5. The maximum of pure eSM hardly changed with T (Fig. 5A), and the same could be said of the Cer-containing mixtures (Fig. 5B,C). This was in contrast with the corresponding vibration band of DPPC, that was shifted abruptly at about 38 °C (Fig. S4A). Our interpretation is that the phosphate group of SM was more firmly anchored by H-bonds than the one of DPPC, hence its increased thermal stability. Addition of Cer would also increase the density of the H-bonding network in the sample, leading to additional stability, and to less noisy T-plots (Fig. 5). In DPPC, addition of 30 mol% Cer shifts the 1091 cm^−1^ band to 1089 cm^−1^ (perhaps due to H-bonding^30^) and abolishes (or widens considerably) the transition in the 10–80 °C range (Fig. S4B).

## Discussion

The involvement of the SM:Cer system in the early stages of the sphingolipid signaling pathway is well established, as is the role of Cer in apoptosis and, under certain conditions, in autophagy^2,35,36^. The acid sphingomyelinase/ceramide system also regulates the internalization of bacteria into the host cell, the subsequent cytokine release, inflammatory response, and initiation of host cell apoptosis^37^. In most, if not all, of these reactive mechanisms Cer is formed as a result of SM cleavage by sphingomyelinase, with the outcome of a system in which both SM and Cer coexist in the cell membrane. Cer hardly mixes with other membrane lipids, with the exception of SM^4^. SM and Cer interact strongly, giving rise to highly rigid gel-like domains, even in the fluid lipid environment of cell membranes^7,12,14,20^.

In general, the overall properties of the SM:Cer domains have been studied in more detail than the molecular interactions between those two molecules, although the latter should be essential for a proper understanding of domain formation. It is generally accepted that sphingolipids establish intermolecular associations not accessible to the glycerolipids because of the extensive H-bond networks that the former can generate^6^. Gillams et al.^8^ described in detail the H-bonds of Cer to water using a combination of experimental (NMR and neutron diffraction techniques) and computational techniques (empirical potential structure refinement and molecular dynamics).

Leung et al.^18^ used D-NMR in combination with selectively deuterated SM or Cer to describe in great detail the acyl-chain interactions between the two lipids. Meanwhile the SM:Cer interactions at the level of the lipid headgroups remain virtually unknown. Boulgaropoulos et al.^19^ published the carbonyl/amide band of mixtures of SM and phosphatidylcholine with and without Cer, but the simultaneous presence of C=O signals arising from both phospholipids and Cer complicated somewhat the analysis. The present paper intends to proceed along this pathway, taking advantage of the fact that IR spectroscopy allows the simultaneous examination of all the chemical groupings in a molecule, namely the polar and non-polar parts in the case under study.

The behaviour of the non-polar acyl chains is coherent with previous studies. C-H stretching, asymmetric (Fig. 1, Fig. S3A,F) and symmetric (Fig. S3B,G), and CH_2_ scissoring (Fig. 2, Fig. S3C,H) vibration bands witness to a thermotropic ordered-to-disordered transition at about 40 °C for pure SM. In the presence of 15 or 30 mol% Cer, the transition is shifted to higher temperatures in a dose-dependent way, by over 30 °C for the 70:30 mixture. This is in good agreement with our previous DSC^7,12^ (Fig. S1) and D-NMR^18^ data. The latter study, using selectively deuterated Cer and SM, allowed the separate observation of the melting of each of the two lipids. The IR data can be interpreted in the framework of the available phase diagrams (Fig. S1B, Figs. 5–7 in ref.^18^). Figure S1B was built on DSC thermograms obtained with eSM, and for both pure eSM and pure pSM the correlation of the 2915–2921 cm^−1^ data with DSC and D-NMR data is very good (± 1 °C) (Table 1). However an important difference between IR and DSC is the T_s_ temperature when Cer is present in the bilayers, it remains virtually unchanged according to DSC, while it increases markedly according with the 2915–2921 cm^−1^ IR data (Fig. 1, Table 1). D-NMR also indicates an increase in T_s,_ particularly for Cer concentrations above 20 mol% (see Fig. 6 in ref.^18^). The explanation resides probably in the different molecular information provided by each technique: both IR and D-NMR report on the average state of molecular order of all the C–H (or CD) bonds, while DSC responds to cooperative changes in hydrocarbon chain heat absorption/release, for which a given number of acyl chains must respond concertedly. The data suggest that, in the presence of Cer, some cooperative units start melting (cooperatively absorbing heat) while the average chains remain in the ordered state. Those early melting cooperative units might well be Cer-rich (micro)domains^12^, that would absorb a sizable amount of heat while remaining a minor population in the sample.

The data in this paper describing the thermal behaviour of bands associated to the lipid headgroups are more novel. The amide band of pure SM exhibits a maximum at 1638 cm^−1^ at room temperature, which is shifted by about 8 cm^−1^ after the thermotropic transition. Note that, unlike the acyl chains, melting appears to begin here at ≈ 30 °C. The presence of Cer complicates the spectrum, because then both amide bands, from SM and Cer, overlap. However, the Cer effect is clear, causing a marked shift of the band towards lower wavenumbers, and changing the overall shape of the band with the appearance of an intense component at ≈ 1630–1640 cm^−1^. There are no signs of the amide band splitting in two in the SM:Cer mixtures, the single band being an indication of the two molecules forming a complex, presumably stabilized by H-bonds. The other spectral component arising from the lipid headgroup is the band with a maximum at 1086 cm^−1^, assigned to asymmetric PO_2_^−^ stretching, and originating purely from SM. The fact that this band does not shift position with temperature, or does it very gradually, at variance with DPPC, suggests that in SM the phosphate group, perhaps the whole polar headgroup, is held in place by H-bonds. The PO_2_^−^ stretching band shift is virtually abolished in the presence of Cer (Fig. 5B,C), perhaps because Cer offers extra H-bond anchoring to the SM headgroup (note that the 1086 cm^−1^ band arises solely from SM).

In summary, IR spectroscopy provides a tool for the simultaneous observation of the polar and nonpolar moieties of lipids in aqueous/D_2_O dispersions. While the hydrophobic acyl chains behave similarly in sphingolipids and glycerolipids, the corresponding polar headgroups differ, due to the extensive H-bond network permitted by the amide group in C2 and hydroxyl in C3 of sphingosine, which lack an equivalent in glycerolipids. In the particular case of SM:Cer mixtures, the IR spectra are indicative of dense intermolecular H-bonding in bilayers, that would in turn be at the origin of the lateral phase separation^38^ that has been associated to platform formation and cell response to stress^2,37^.

## Supplementary information

Supplementary Information.
