# Supplementary material for: Exploring polar headgroup interactions between sphingomyelin and ceramide with infrared spectroscopy
Source: Sci Rep. 2020 Oct 19;10:17606. doi: 10.1038/s41598-020-74781-8 (PMC7573612; doi:10.1038/s41598-020-74781-8)

**SUPPLEMENTARY MATERIAL**

**Exploring polar headgroup interactions between sphingomyelin and ceramide with infrared spectroscopy**

Igor de la Arada, Emilio J. González-Ramírez, Alicia Alonso, Félix M. Goñi*, José-Luis R. Arrondo

Instituto Biofisika (CSIC, UPV/EHU), and Departamento de Bioquímica, Universidad del País Vasco, 48940 Leioa, Spain.

**Supplementary figure legends.**

**Fig. S1. Differential scanning calorimetry of aqueous dispersions of pure eSM and eSM:Cer mixtures. (**A) Calorimetric traces. Ceramide concentration in the mixtures is indicated for each thermogram. (B) Partial phase diagram for the eSM:Cer system in excess water, derived from DSC measurements. Figure reproduced from Sot et al. (2006) with permission.

**Fig. S2. Selected regions in the IR spectra of pure eSM and eSM:Cer dispersions in excess D_2_O.** Top, pure eSM. Bottom, eSM:Cer (70:30). A, E, asymmetric C-H stretching vibrations. B, F, CH_2_ scissoring vibrations. C, G, amide I band. D, H, phosphate group vibrations. Continuous line, 20ºC. Discontinuous line, 80ºC.

**Fig. S3. Temperature plots of the IR band positions in SM spectra.** Top, eSM. Bottom, pSM. A, F, asymmetric C-H stretching vibrations. B, G, symmetric C-H stretching vibrations. C, H, CH_2_ scissoring vibrations. D, I, amide I band. E, J, phosphate group vibrations.

**Fig. S4.** **Temperature plot of the 1086 cm^-1^ PO_2_^-^ band positions of DPPC spectra in excess D_2_O.**

**Fig. S5. Temperature plots of the 1086 cm^-1^ PO_2_^-^ band intensities of pure eSM and eSM:Cer dispersions in excess D_2_O.** Intensities are absorbances measured at the band maxima.

**Fig. S1**

**Fig. S2**

**
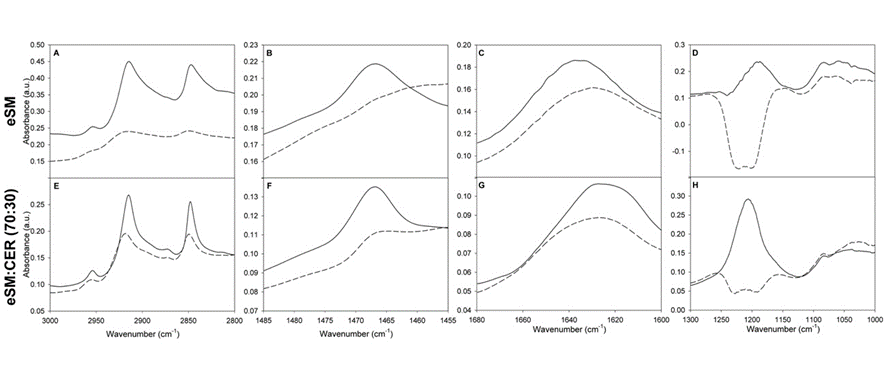
**

**Fig. S3**

**
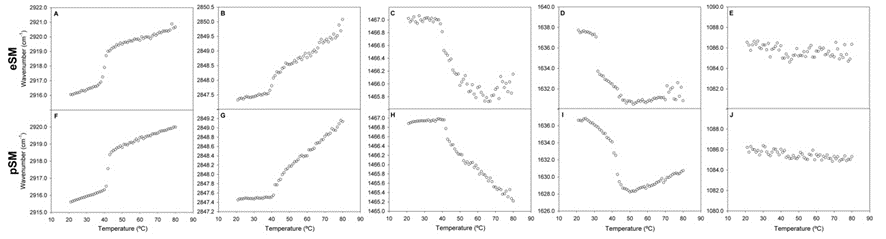
**

**Fig. S4**

**
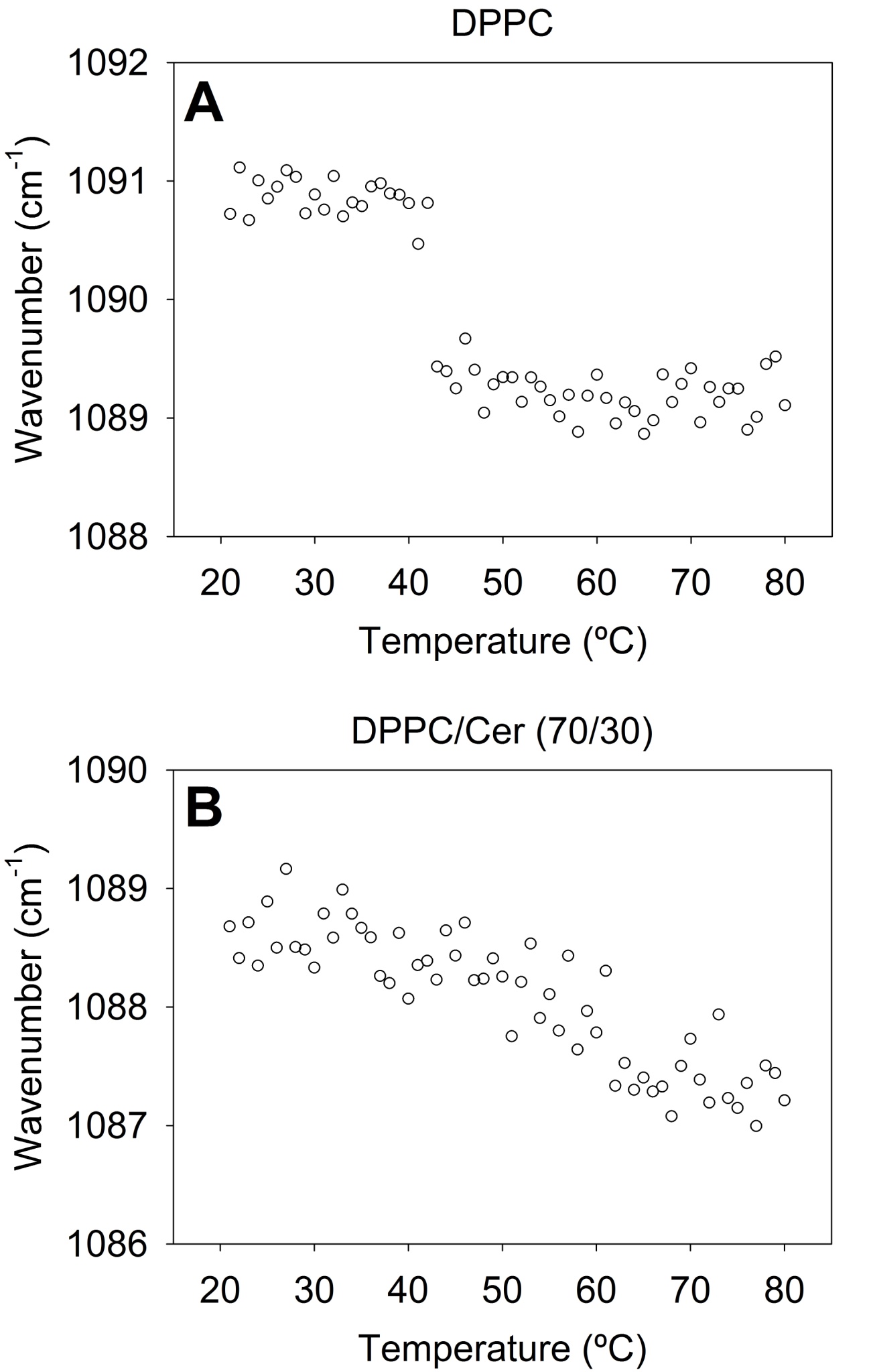
**

**Fig. S5**


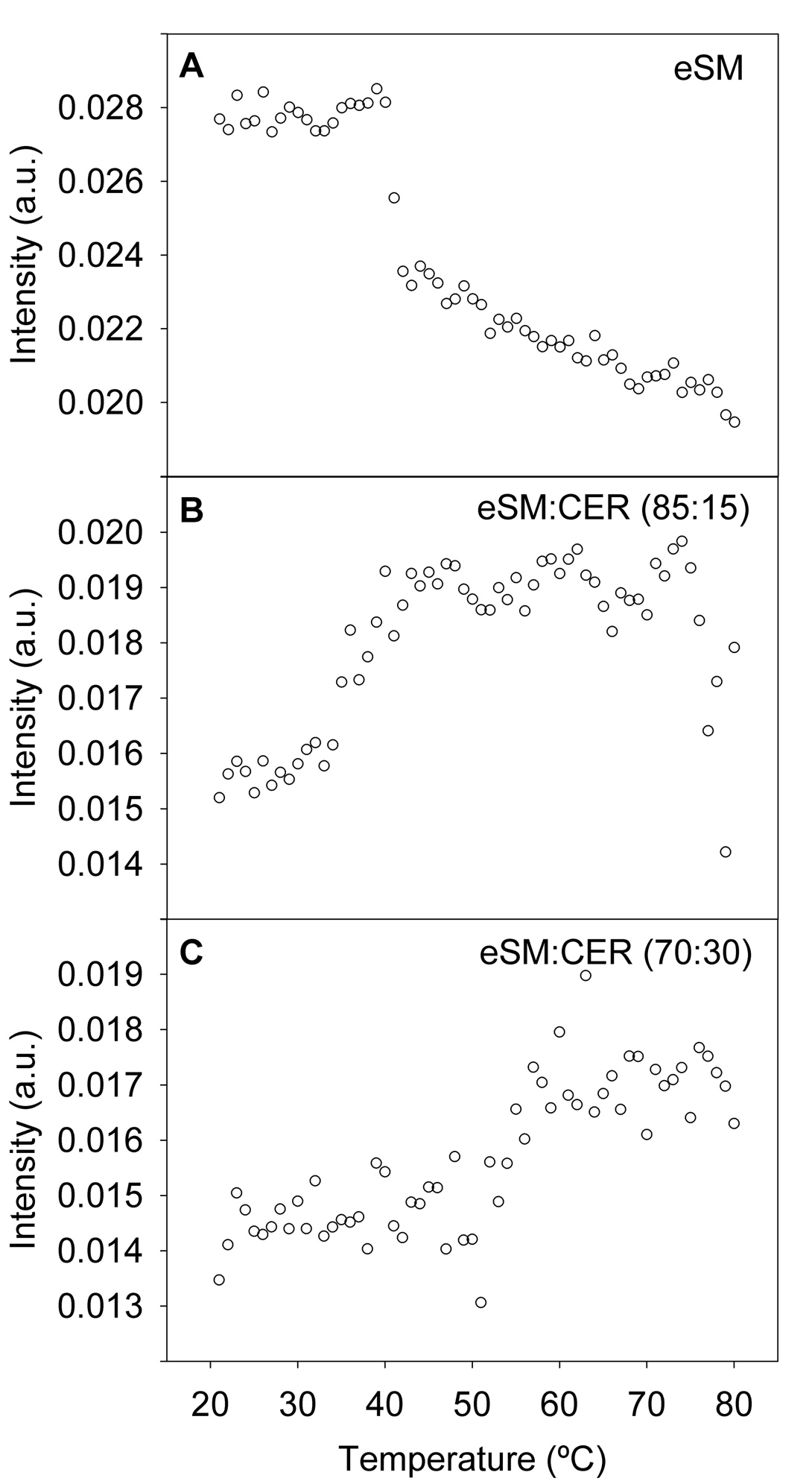

Supplement: Supplementary file 1 — Supplementary Information. [file 41598_2020_74781_MOESM1_ESM.docx]
